# Effect of Salt Substitution on Ambulatory Blood Pressure, Kidney Function and Inflammation in Middle-Aged and Elderly Hypertensive Patients

**DOI:** 10.31083/j.rcm2505158

**Published:** 2024-05-08

**Authors:** Li Che, Jiayu Fu, Ying Zhang, Yunpeng Cheng, Yan Liu, Wei Song, Yinong Jiang

**Affiliations:** ^1^Department of Cardiology, Central Hospital of Dalian University of Technology, 116033 Dalian, Liaoning, China; ^2^Yingkou Central Hospital, 115003 Yingkou, Liaoning, China; ^3^Department of Cardiology, First Affiliated Hospital of Dalian Medical University, 116011 Dalian, Liaoning, China

**Keywords:** ambulatory blood pressure, salt substitution, kidney, inflammation

## Abstract

**Background:**

Low-sodium (LS) salt substitution is recognized for its potential to reduce blood pressure (BP), but most research relies on office BP measurement (OBPM). There is a lack of data on salt substitution’s effect on target organs, such as the kidney as measured by the urine albumin-to-creatinine ratio (UACR), and its impact on inflammatory cytokines, particularly high-sensitivity C-reactive protein (hs-CRP). To evaluate the effect of LS salt substitution on ambulatory BP measurement (ABPM), kidney function, and inflammation in middle-aged and elderly hypertensive patients.

**Methods:**

In this 12-month prospective, multi-center, randomized, double-blind study, 352 hypertensive patients were randomly assigned to the normal salt (NS) group (n = 176) or the LS group (n = 176) at a 1:1 ratio. ABPM, fasting blood, and morning first spot urine samples were obtained at baseline and the endpoint.

**Results:**

Of the 352 patients, 322 completed all follow-up surveys, and 301 underwent ABPM. In the LS roup, significant reductions were observed in 24-hr systolic BP (–2.3 mmHg), 24-hr diastolic BP (–1.5 mmHg), daytime systolic BP (–2.6 mmHg), daytime diastolic BP (–1 mmHg), and nighttime systolic BP (–0.1 mmHg) compared to the NS group (all *p* < 0.05). However, the change in nighttime diastolic BP was not statistically significant (–0.3 vs. 1.1 mmHg, *p* = 0.063). Additionally, the LS group showed a more substantial decrease in UACR (–2.05 vs. –7.40 µg/mg, *p* = 0.004) and hs-CRP (–0.06 vs. –0.24 mg/L, *p* = 0.048) compared to NS.

**Conclusions:**

LS salt substitution significantly decreased ABPM, suggesting a notable impact on hypertension. Furthermore, it demonstrated a protective impact on kidney function, as evidenced by changes in UACR. Additionally, LS salt substitution appeared to reduce inflammation, indicated by the decrease in hs-CRP levels.

**Clinical Trial Registration:**

The study was registered in the Chinese clinical trial registry (registration number: ChiCTR1800019727).

## 1. Introduction

Hypertension significantly increases the risk of stroke and ischemic heart disease, leading to higher morbidity and mortality rates [1]. Effective blood pressure (BP) management is crucial to mitigating the risks of cardiovascular disease and death [2]. In China, while 23.2% of adults suffer from hypertension and 41.3% from pre-hypertension, the BP control rate remains low at 15.3%, even with the use of various efficient antihypertensive agents [3]. Some studies have shown that BP increased after salt loading [4] and decreased after salt restriction [5]. Other studies indicated that potassium replenishment was linked to BP reduction [6]. Hence, non-pharmacological treatments, such as salt restriction and potassium supplementation, are recommended to enhance the effect of antihypertensive drugs [7]. Studies have confirmed that reducing sodium and increasing potassium intake can lower BP [8, 9], particularly in hypertensive individuals [10]. However, the validity of these findings is somewhat limited, as most prior trials relied on office BP measurement (OBPM). Compared to OBPM, ambulatory BP measurement (ABPM) provides 24-hour BP values, offering advantages in cost-effectiveness, accurate hypertension diagnosis, avoiding white-coat hypertension [7] and better prediction of target organ damage and cardiovascular disease [11].

Currently, it is unclear how low-sodium (LS) salt substitution affects target organs, particularly the kidneys, as measured by the urine albumin-to-creatinine ratio (UACR). Additionally, the impact of LS salt substitution on inflammatory cytokines, such as high-sensitivity C-reactive protein (hs-CRP), remains unexplored. Given that UACR is a recognized indicator of kidney disease progression [12] and rising hs-CRP levels correlate with increased blood pressure and various cardiovascular risks [13], understanding the effects of salt substitution on these markers is crucial.

Our study, therefore, seeks to assess the efficacy of LS salt substitution in reducing blood pressure via ABPM among middle-aged and elderly hypertensive patients in northern China. This research aims to provide dietary strategies for hypertension prevention and treatment. Additionally, we aim to determine the impact of salt substitution on kidney health and hs-CRP levels.

## 2. Methods

### 2.1 Participants

We recruited hypertensive patients from August, 2019 from two community centers in Dalian City, Liaoning Province, China. Hypertensive status was defined as systolic BP (SBP) ≥140 mmHg and/or diastolic BP (DBP) ≥90 mmHg or taking antihypertensive agents [7]. Inclusion criteria were: (1) primary hypertensives with 140 ≤ SBP ≤ 180 mmHg and/or 90 ≤ DBP ≤ 110 mmHg measured by professional doctors; (2) aged ≥50 and ≤70 years; (3) at least had two meals at home per day; (4) had no severe renal insufficiency; (5) baseline serum potassium concentration lower than 5.0 mmol/L.

### 2.2 Study Design

This 12-month study utilized a computerized randomization program to divide participants into two groups in a 1:1 ratio into the normal salt (NS) group or the LS group. The NS was comprised of 100% sodium chloride, while the LS substitution contained 43% sodium chloride, 32% potassium chloride and 25% other ingredients.

### 2.3 Demographic Characteristics

**Fig. 1.**
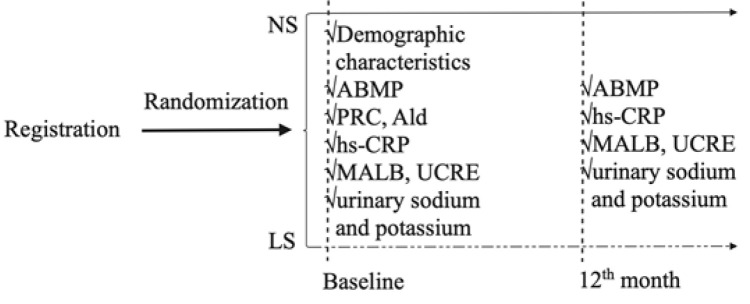
**Flow chart of the study**. NS, normal salt; LS, low-sodium; ABPM, ambulatory blood pressure measurement; PRC, plasma renin concentration; Ald, aldosterone; hs-CRP, high-sensitivity C-reactive protein; MALB, microalbuminuria; UCRE, urine creatinine.

Fig. 1 presents the flow chart of the study. The baseline investigation included interviews of all participants conducted by professional doctors to gather demographic characteristics from all participants.

### 2.4 Ambulatory Blood Pressure Measurement

Baseline and endpoint ABPM were taken in the right arm using a validated electronic upper-arm cuff device (ABPM50, Qinhuangdao, China). Daytime measurements were taken at 30-min intervals from 6:00 AM to 10:00 PM; nighttime measurements were taken at 60-min intervals from 10:00 PM to 6:00 AM. Patients were informed to continue their daily activities but avoid vigorous activity. At least 20 valid daytime and 7 nighttime BP readings were required for the measurements to be considered successful. If not, participants were scheduled to take the exam again [7], and they were excluded if there were insufficient valid readings.

### 2.5 Biochemistry

Fasting blood samples were collected to measure plasma renin concentration (PRC), aldosterone (Ald), and hs-CRP along with first morning urine samples for measuring microalbuminuria (MALB), urine creatinine (UCRE), and spot urinary sodium and potassium, the latter to ensure compliance with the LS salt substitution regimen [14]. These samples were obtained from each participant at baseline and at the study’s endpoint. Subsequently, all blood and urine specimens were sent to the clinical laboratory of Dalian Medical University (DMU) for analysis.

### 2.6 Statistics

We used the following formula to calculate the sample size:

$$N=\left(1+1/\kappa \right)\,{\left(\sigma \,\frac{\mathrm{z1}-\frac{\alpha }{2}+\mathrm{z1}-\beta }{\mu \,A-\mu \,B}\right)}^{2}$$

the value of α was 0.05 and β was 0.1. The minimal sample size was 160 in both groups. This number was increased by 10% to compensate for potential losses due to follow-up or other reasons. Hence, the final sample size was 352 patients.

We conducted statistical analyses using SPSS 25.0 (IBM Corp., Armonk, NY, USA). We reported data as mean ± SD, and *t*-tests were used to compare continuous variables with a normal distribution. For variables with an abnormal distribution, median and inter-quartile ranges were used, and the Mann–Whitney U test was applied for comparisons. Frequencies were used to describe categorical variables, and the Chi-squared test was employed for comparisons. A *p* value of <0.05 was considered statistically significant.

## 3. Results

### 3.1 Demographic Characteristics of the Participants

Initially, 352 participants were enrolled in the study. Of these, 30 (8.5%) dropped out, leaving 322 who completed all follow-up visits, and 301 who completed the ABPM. The average participant age was 62.17 ± 4.69 years in the NS group and 62.96 ± 4.51 years in the LS group. There were 72 (45.0%) male subjects in the NS group and 58 (35.8%) in the LS group. No significant differences were recorded in baseline age, sex, body mass index (BMI), and antihypertensive medication (all *p* > 0.05) (Table 1).

**Table 1.** **Baseline demographic characteristics of the participants**.

Characteristics		NS	LS	*p*
		(n = 156)	(n = 145)	
Age (y)		62.3 ± 4.7	63 ± 4.6	0.161
Male sex (n (%))		70 (44.9)	50 (34.5)	0.066
BMI (kg/$m^{2}$)		25.6 ± 3.1	26 ± 3.3	0.238
Smoke history (n (%))		25 (16)	24 (16.6)	0.902
Alcohol history (n (%))		20 (12.8)	15 (10.3)	0.503
Medication				
	CCB (n (%))	105 (67.3)	98 (67.6)	0.959
	ACEI/ARB (n (%))	58 (37.2)	61 (42.1)	0.386
	β-blocker (n (%))	26 (16.7)	20 (13.8)	0.489
	ACEI/ARB + CCB (n (%))	27 (17.3)	36 (24.8)	0.109
	β-blocker + CCB (n (%))	19 (12.2)	16 (11)	0.757
	β-blocker + ACEI/ARB (n (%))	9 (5.8)	11 (7.6)	0.527
	β-blocker + CCB + ACEI/ARB (n (%))	5 (3.2)	10 (6.9)	0.141
PRC (uIU/mL)		11.9 (5.2, 24.4)	11.2 (4, 22.1)	0.355
Ald (pg/mL)		105 (73, 152.8)	97.8 (68, 147)	0.580
ARR		8.3 (4, 17.8)	9.6 (4.1, 23.8)	0.391

NS, normal salt; LS, low-sodium; BMI, body mass index; CCB, calcium channel blocker; ACEI, angiotensin-converting enzyme inhibitor; ARB, angiotensin II receptor blocker; PRC, plasma renin concentration; Ald, aldosterone; ARR, aldosterone/renin ratio.

### 3.2 Comparison of ABPM at Baseline and the Endpoint

At baseline, there were no significant differences in ABPM between the two groups for any period (all *p* > 0.05). However, at the study endpoint, there were noteworthy changes in blood pressure levels. The 24-hour diastolic blood pressure (24hDBP) was significantly lower in the LS group when compared to the NS group (*p* = 0.008). Additionally, there was a non-significant trend towards a decrease in the 24-hr systolic BP (24hSBP) compared to the NS group.

**Fig. 2.**
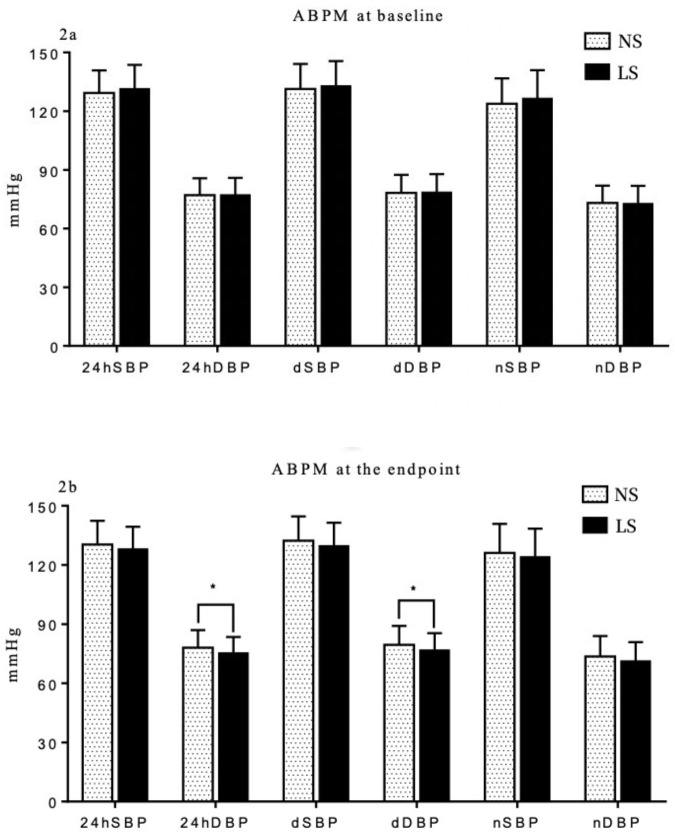
**Comparison of ambulatory BP measurement (ABPM) between NS and LS group at baseline (2a) and the endpoint (2b)**. * *p* < 0.05. NS, normal salt; LS, low-sodium; SBP, systolic blood pressure; DBP, diastolic blood pressure; d, daytime; BP, blood pressure; n, nighttime.

The daytime DBP (dDBP) was significantly lower in the LS group (*p* = 0.015). The daytime SBP (dSBP) also exhibited a non-significant trend towards a decrease in the LS group. Despite the presence of a trend towards a decrease, neither the nighttime SBP (nSBP) or nighttime DBP (nDBP) reached statistical significance (Fig. 2).

### 3.3 Comparison of ABPM Changes (Endpoint Minus Baseline)

Examining the difference from baseline yielded notable results as well. We found significant differences in both Δ24hSBP (*p* = 0.004) and Δ24hDBP (*p* = 0.007) in the LS cohort when compared to the NS cohort. Additionally, ΔdSBP (*p* = 0.012), ΔdDBP (*p* = 0.012), and ΔnSBP (*p* = 0.007) differed significantly as well. In contrast, there were no differences between groups when assessing ΔnDBP (Table 2).

**Table 2.** **Comparison of ABPM changes (endpoint minus baseline) between NS and LS group**.

	NS	LS	*p*
Δ24hSBP (mmHg)	0.6 (–6.2, 6.8)	–2.3 (–7.8, 3.5)	0.004*
Δ24hDBP (mmHg)	0.1 (–3.6, 6.1)	–1.5 (–6, 2.7)	0.007*
ΔdSBP (mmHg)	–0.3 (–6.8, 7.5)	–2.6 (–8.9, 4.5)	0.012*
ΔdDBP (mmHg)	0.7 (–5.0, 7.5)	–1 (–7.2, 3.8)	0.012*
ΔnSBP (mmHg)	2.6 (–6.4, 10.2)	–0.1 (–10.0, 6.3)	0.007*
ΔnDBP (mmHg)	1.1 (–5.8, 5.4)	–0.3 (–6.9, 4.4)	0.063

Δ, net difference; *p < 0.05. NS, normal salt; LS, low-sodium; SBP, systolic blood pressure; DBP, diastolic blood pressure; n, nighttime; d, daytime; ABPM, ambulatory blood pressure measurement.

### 3.4 Comparison of Changes in ABPM for Different Subgroups according to Baseline PRC, Aldosterone Concentration, and Aldosterone/Renin Ratio (ARR)

**Fig. 3.**
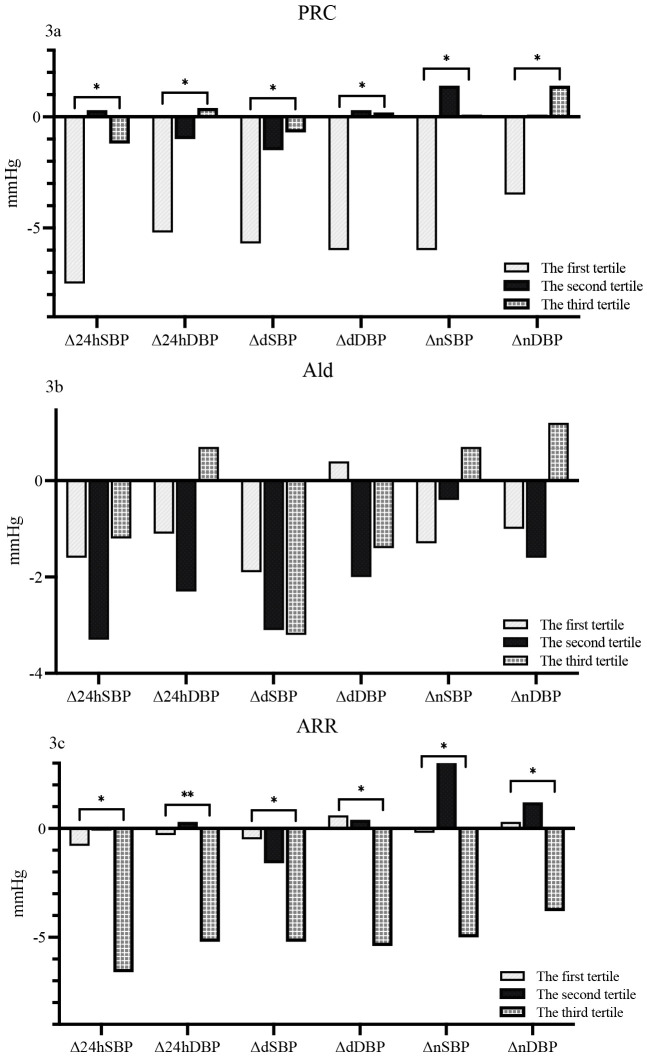
**Comparison of changes in ABPM for different subgroups according to baseline PRC (3a), aldosterone concentration (3b) and ARR (3c)**. Δ, net difference; * *p* < 0.05; ** *p* < 0.001; Ald, aldosterone; ARR, aldosterone/renin ratio; PRC, plasma renin concentration; SBP, systolic blood pressure; DBP, diastolic blood pressure; n, nighttime; d, daytime.

Participants were divided into tertiles based on their PRC. In the first tertile of the PRC subgroup (PRC: ≤5.896 uIU/mL), there was a notable decrease in 24hSBP and 24hDBP by –7.5 mmHg and –5.2 mmHg, respectively. In the second tertile (PRC: 5.896–17.040 uIU/mL) the changes were minimal at 0.3/–1 mmHg for 24hSBP and 24hDBP. For the third tertile (PRC: >17.040 uIU/mL) the changes were –1.2/0.4 mmHg. Daytime values for dSBP and dDBP also varied across tertiles. In the first, second, and third tertiles, the changes were –5.7/–6 mmHg, –1.5/0.3 mmHg, and –0.7/0.2 mmHg, respectively. Nighttime systolic and diastolic blood pressure (nSBP and nDBP) followed a similar pattern, with changes of –6/–3.5 mmHg in the first tertile, 1.4/0.1 mmHg in the second, and 0.1/1.4 mmHg in the third. Comparatively, blood pressure changes were more pronounced in the low PRC group than in the high PRC group. This difference was statistically significant for all measurements: 24hSBP (*p* = 0.001), 24hDBP (*p* = 0.003), dSBP (*p* = 0.011), dDBP (*p* = 0.006), nSBP (*p* = 0.003), and nDBP (*p* = 0.006) as shown in Fig. 3a.

In the first tertile of aldosterone subgroups (Ald: ≤84.000 pg/mL), changes in 24-hr BP were modest, at –1.6/–1.1 mmHg for systolic and diastolic pressures, respectively. The second tertile (Ald: 84.000–130.000 pg/mL) showed slightly greater changes at –3.3/–2.3 mmHg. However, in the third tertile (Ald >130.000 pg/mL) the changes were –1.2/0.7 mmHg, indicating lesser change in systolic and an increase in diastolic pressures. Changes in daytime BP followed a similar pattern, with the first, second, and third tertiles showing changes of –1.9/0.4 mmHg, –3.1/–2 mmHg, and –3.2/–1.4 mmHg, respectively. For nighttime BP, the changes were –1.3/–1 mmHg in the first tertile, slightly different at –0.4/–1.6 mmHg in the second, and 0.7/1.2 mmHg in the third, indicating an increase in both systolic and diastolic pressures for the highest aldosterone subgroup. Despite these variations, no significant differences were observed between the three subgroups for any of the blood pressure changes (*p* = 0.643 for 24hSBP, *p* = 0.184 for 24hDBP, *p* = 0.622 for dSBP, *p* = 0.127 for dDBP, *p* = 0.401 for nSBP, and *p* = 0.165 for nDBP), as illustrated in Fig. 3b.

In the first tertile of the ARR subgroup (ARR: ≤5.420), 24-hr BP changes were minimal, at –0.8/–0.3 mmHg for systolic and diastolic pressures, respectively. In the second tertile (ARR: 5.420–16.550) the changes were even more modest at –0.1/0.3 mmHg. However, the third tertile (ARR: >16.550) exhibited substantial changes, with reductions of –6.6/–5.2 mmHg. Daytime dBP changes also varied across tertiles. The first, second, and third tertiles showed changes of –0.5/0.6 mmHg, –1.6/0.4 mmHg, and –5.2/–5.4 mmHg, respectively. Nighttime nBP changes followed a similar trend, with the first and second tertiles showing minimal changes of –0.2/0.3 mmHg and 3.1/1.2 mmHg, respectively, and the third tertile showing a significant reduction of –5/–3.8 mmHg. Notably, BP changes in the high ARR group were significantly greater than those in the low ARR group. This was evident across all measurements (*p* = 0.001 for change in 24hSBP, *p* < 0.001 for change in 24hDBP, *p* = 0.012 for change in dSBP, *p* = 0.001 for change in dDBP, *p* = 0.008 for change in nSBP, and *p* = 0.006 for change in nDBP) (Fig. 3c).

### 3.5 MALB, UCRE and UACR at Baseline and the Endpoint

At baseline, there were no significant differences were found between the two groups in terms of MALB, UCRE, and UACR. By the study endpoint, notable differences emerged. Specifically, the UACR was significantly lower participants assigned to the LS cohort, with median values of 12.48 µg/mg (interquartile range [IQR]: 5.12, 26.46) vs. 19.63 µg/mg (IQR: 7.65, 36.94) and a p value of 0.026 indicating statistical significance. Meanwhile, no significant differences were observed in MALB and UCRE between the two groups at the endpoint (Table 3).

**Table 3.** **MALB, UCRE, UACR at baseline and the endpoint**.

	Baseline			Endpoint		
	NS	LS	*p*	NS	LS	*p*
MALB (mg/L)	21.96 (13.55, 40.31)	20.76 (12.94, 38.32)	0.630	14.11 (7.12, 30.51)	12.33 (1.97, 22.96)	0.148
UCRE (mmol/L)	11.58 (7.9, 13.9)	11 (7.92, 13.98)	0.478	6.78 (4.51, 9.61)	6.94 (4.1, 10.29)	0.545
UACR (µg/mg)	17.48 (11.5, 29.63)	17.25 (10.54, 32.79)	0.830	19.63 (7.65, 36.94)	12.48 (5.12, 26.46)	0.026*

*p < 0.05. NS, normal salt; LS, low-sodium; MALB, microalbuminuria; UCRE, urine creatinine; UACR, urine albumin-to-creatinine ratio.

### 3.6 Comparison of MALB, UCRE and UACR Changes (Endpoint Minus Baseline) between NS and LS Groups

There were no differences in the changes of MALB and UCRE in the two groups. In contrast, we did observe a significant difference in the UACR change between groups (*p* = 0.004) (Table 4).

**Table 4.** **Comparison of MALB, UCRE and UACR changes (endpoint minus baseline) between NS and LS groups**.

	NS	LS	*p*
ΔMALB (mg/L)	–9.80 (–20.30, 4.09)	–12.50 (–25.69, 1.42)	0.244
ΔUCRE (mmol)	–4.26 (–7.73, –0.45)	–3.32 (–7.25, 0.84)	0.231
ΔUACR (µg/mg)	–2.05 (–12.47, 10.72)	–7.40 (–16.89, 2.01)	0.004*

* *p* < 0.05. NS, normal salt; LS, low-sodium; MALB, microalbuminuria; UCRE, urine creatinine; UACR, urine albumin-to-creatinine ratio; Δ, net difference.

### 3.7 Hs-CRP at Baseline and the Endpoint and Comparison of the Change of hs-CRP (Endpoint Minus Baseline) between the NS and LS Groups

**Fig. 4.**
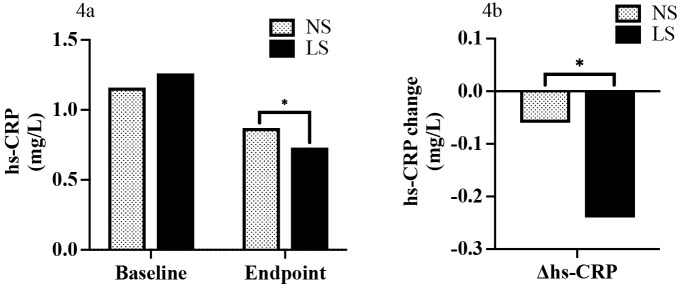
**Hs-CRP at baseline and the endpoint (4a) and comparison of change of hs-CRP (endpoint to baseline) between NS and LS group (4b)**. * *p* < 0.05. NS, normal salt; LS, low-sodium; Δ, net difference; hs-CRP, high-sensitivity C-reactive protein.

At baseline, we found no differences in hs-CRP between the NS and LS groups (1.16 mg/L [IQR: 0.56, 2.61] vs. 1.26 mg/L [IQR: 0.61, 2.2], *p* = 0.999). In contrast, a reduction in hs-CRP was observed at the study endpoint (0.73 mg/L [IQR: 0.40, 1.38] vs. 0.87 mg/L [IQR: 0.52, 1.79], *p* = 0.045 (Fig. 4a). This effect was also found when assessing hs-CRP change (–0.24 mg/L [IQR: –0.96, 0.13] vs. –0.06 mg/L [IQR: –0.93, 0.36], *p* = 0.048) (Fig. 4b).

## 4. Discussion

In our study, we observed that LS salt substitution led to a significant decrease in 24hSBP and 24hDBP, dSBP and dDBP, nSBP in middle-aged and elderly hypertensive patients over a one-year treatment period. However, the decrease in nDBP was not significant.

These findings are in partial alignment with previous research. For instance, a previous study conducted on 20 elderly hypertensive patients found that after using a mineral salt blend (57% NaCl, 28% KCl, 12% ${\mathrm{MgSO}}_{4}$${\mathrm{7H}}_{2}$O), 9 patients experienced significant reductions in 24hSBP and 24hDBP, but no differences were observed in the remaining 11 patients [15]. The overall antihypertensive effect of mineral salt in all 20 patients was not conclusively determined. Another study including only 13 subjects indicated a diet low in sodium and high in potassium led to significant reductions in 24hSBP and 24hDBP after 21 days of intervention [16]. However, the change in nDBP in our study was not statistically significant. One possible reason is that DBP declines less than SBP after salt restriction [17, 18]. Additionally, the lower nighttime urination rate results in reduced sodium and volume excretion during these hours. Our use of ABPM provided more detailed and precise BP values compared with OBPM and home blood pressure measurement (HBPM). This may help to provide accurate treatment protocols for hypertension.

A previous study indicated a positive correlation between increased dietary salt intake and MALB in patients with type 1 diabetes, although potassium did not demonstrate a similar relationship [19]. In our study, we were unable to replicate this finding. This discrepancy may be attributed to variations in the participant populations of the different studies. However, we noticed significant UACR changes in the LS group. The reason may be that UACR is considered a more sensitive and accurate marker for detecting minor changes in MALB [20]. Researchers also found that paranormal levels of UACR were related to adverse events; for example, UACR 1.1 mg/mmol or more was considered an independent predictor for mortality [21]. Based on these findings, we propose that a low-sodium diet may exert a protective effect on kidney function.

Cross-sectional studies have consistently demonstrated a positive correlation between hs-CRP and sodium intake [22]. Interestingly, one study involving 41 hypertensive individuals determined that hs-CRP significantly increased after three weeks of sodium restriction (Na: from 160 to 60 mmol/L per day) [23]. This finding appears to contradict our results, where hs-CRP decreased following LS treatment. The discrepancy could be attributed to the smaller participant sample and shorter duration of the previous study.

As was stated in our previous report, participants assigned to the LS group showed significantly lower urine sodium levels and higher potassium levels at the study’s conclusion compared to the NS group [24]. This outcome not only confirms the effectiveness of the LS salt substitution but also indicates good compliance with the dietary intervention among the participants.

## 5. Conclusions

Our study demonstrated that LS salt substitution plays a significant role in managing hypertension, as evidenced by the marked decrease in ABPM values in middle-aged and elderly hypertensive patients. The substantial reductions in both 24-hour systolic and diastolic blood pressures, as well as daytime and nighttime blood pressures, underline the potential of LS salt substitution as a straightforward, non-pharmacological strategy for effective blood pressure control.

Moreover, the LS intervention exhibited a protective effect on renal function. Although the changes in MALB did not reach statistical significance, the significant decrease in UACR suggests that LS salt substitution may help mitigate the progression of kidney damage, particularly in those at risk of or currently experiencing hypertension. This is of considerable clinical importance, given the UACR’s sensitivity and accuracy in detecting early signs of renal impairment.

Additionally, our findings suggest a potential anti-inflammatory effect of LS salt substitution. Despite some studies indicating a positive relationship between sodium intake and inflammatory markers like hs-CRP, our research observed a decrease in hs-CRP levels following LS treatment. This indicates that reducing sodium intake might also contribute to lowering systemic inflammation, which is a known risk factor for various cardiovascular and renal diseases.

In light of these results, LS salt substitution emerges as a multifaceted dietary approach with the potential to not only lower blood pressure but also confer renal protection and reduce inflammation. It represents a promising avenue for the non-pharmacological management of hypertension and its associated complications. Future studies with larger populations and longer follow-up periods are warranted to further elucidate the long-term benefits and mechanisms of LS salt substitution in diverse populations.

## 6. Limitations

While our study provides valuable insights into the effects of LS salt substitution on blood pressure and renal function, several limitations warrant consideration. First and foremost, our reliance on spot urine samples instead of 24-hour collections might have affected the precision of our measurements for MALB, UCRE, and UACR. While spot samples are more convenient, 24-hour urine collections are considered the gold standard for assessing renal excretion and function, offering a more comprehensive overview of renal health and the body’s sodium handling. Therefore, the accuracy of our renal-related findings might be somewhat compromised.

Second, not all participants who completed the follow-up visits underwent ABPM. ABPM is a critical component in accurately assessing the impact of LS salt substitution on blood pressure, providing detailed insights into the variations over a 24-hour period. The lack of ABPM data for all participants may limit the generalizability of our blood pressure findings and potentially mask the full extent of the benefits or risks associated with LS salt substitution.

Additionally, while our study had a reasonable follow-up period, longer-term studies are necessary to fully understand the sustained effects and potential long-term benefits or drawbacks of LS salt substitution. Changes in kidney function and blood pressure may evolve over years, and a longer observation period would provide a more robust assessment of the intervention’s efficacy and safety.

It’s also worth noting that our study population was limited to middle-aged and elderly hypertensive patients in a specific geographic region, the Liaoning Province of China. The findings might not be universally applicable to other age groups, ethnicities, or individuals with different dietary habits and health statuses. Moreover, dietary compliance and the precise quantification of salt intake were not directly monitored, which could introduce variability in the actual sodium and potassium intake among participants.

In conclusion, while our study presents promising findings regarding the benefits of LS salt substitution, these limitations highlight the need for further research. Future studies employing 24-hour urine collections, ensuring complete ABPM data for all participants, extending the follow-up duration, and including a more diverse population would help validate and extend our conclusions.

## Data Availability

The data sets generated and analyzed during the current study are not publicly available but are available from the corresponding author on reasonable request.
